# The neonate respiratory microbiome

**DOI:** 10.1111/apha.14266

**Published:** 2025-01-22

**Authors:** Sabine Pirr, Maike Willers, Dorothee Viemann

**Affiliations:** ^1^ Department of Pediatric Pneumology, Allergology and Neonatology Hannover Medical School Hannover Germany; ^2^ Cluster of Excellence RESIST 2155—Resolving Infection Susceptibility, Hannover Medical School Hannover Germany; ^3^ University Hospital Freiburg PRIMAL (Priming Immunity at the Beginning of Life) Consortium Freiburg Germany; ^4^ Translational Pediatrics, Department of Pediatrics University Hospital Würzburg Würzburg Germany; ^5^ Center for Infection Research University Würzburg Würzburg Germany

**Keywords:** gut–lung axis, host–immunity interaction, microbiome development, microbiota, neonate, respiratory disease, respiratory tract

## Abstract

Over the past two decades, it has become clear that against earlier assumptions, the respiratory tract is regularly populated by a variety of microbiota even down to the lowest parts of the lungs. New methods and technologies revealed distinct microbiome compositions and developmental trajectories in the differing parts of the respiratory tract of neonates and infants. In this review, we describe the current understanding of respiratory microbiota development in human neonates and highlight multiple factors that have been identified to impact human respiratory microbiome development including gestational age, mode of delivery, diet, antibiotic treatment, and early infections. Moreover, we discuss to date revealed respiratory microbiome–disease associations in infants and children that may indicate a potentially imprinting cross talk between microbial communities and the host immune system in the respiratory tract. It becomes obvious how insufficient our knowledge still is regarding the exact mechanisms underlying such cross talk in humans. Lastly, we highlight strong findings that emphasize the important role of the gut–lung axis in educating and driving pulmonary immunity. Further research is needed to better understand the host – respiratory microbiome interaction in order to enable the translation into microbiome‐based strategies to protect and improve human respiratory health from early childhood.

## 1 INTRODUCTION

**FIGURE 1:**
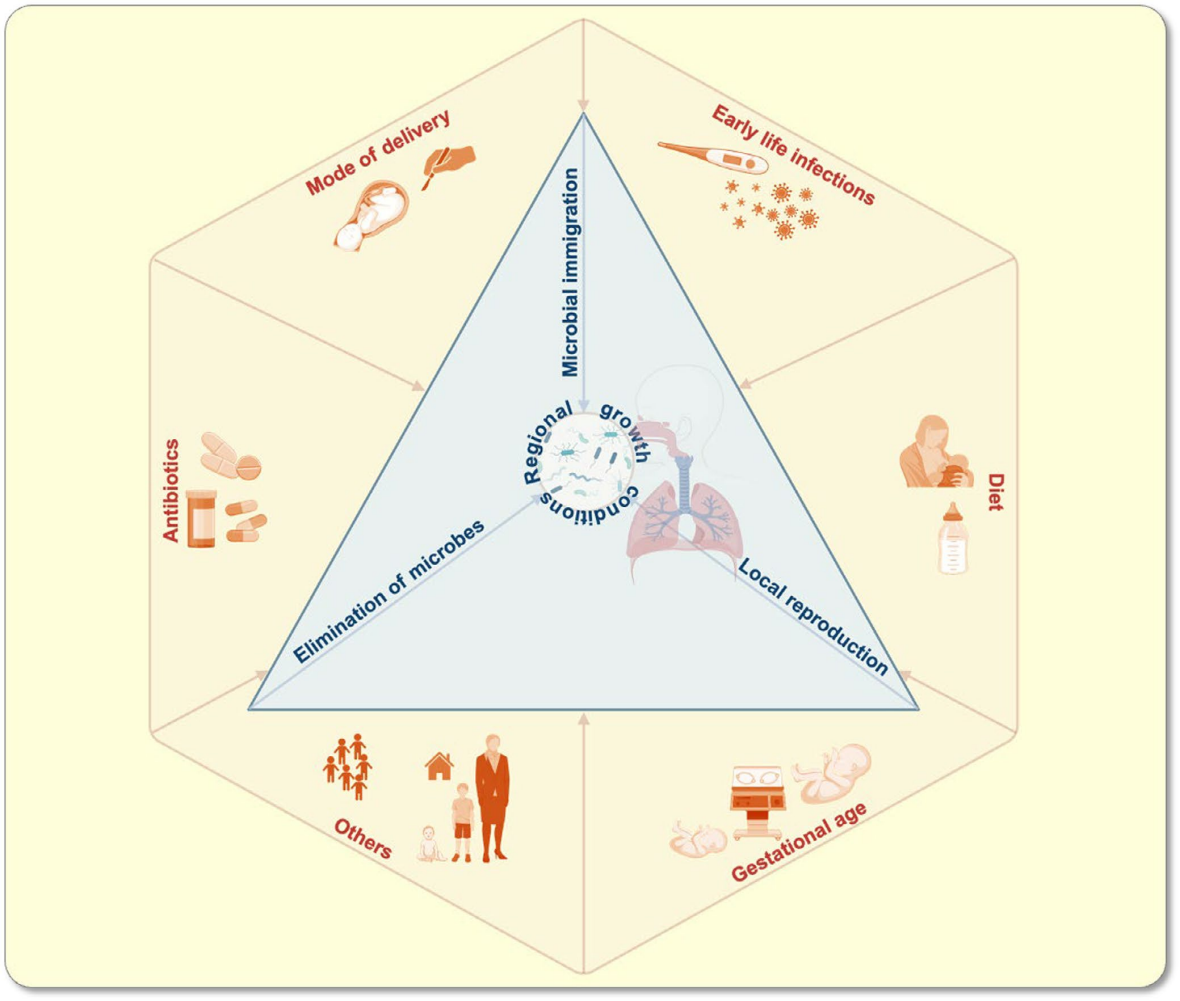
Ecological processes impacting on the respiratory microbiome (inner triangle) and factors influencing these processes during the development of the respiratory microbiome (outer hexagon).

The human body provides a broad range of niches for a vast number of microbes to live in and is considered home of the most complex microbial ecosystem on this planet. The microorganisms that colonize the multiple surfaces of the human body, including skin, oral cavity, respiratory tract, urogenital, and gastrointestinal tract, represent the so‐called human microbiota.^1^ Each of these microbial communities occupying a particular organ with its set of physicochemical properties, including its collective genome and gene products, defines a microbiome.^2^, ^3^ Over the past two decades, it has become clear that the composition and function of the human microbiome have a critical role in the health of infants, young children, and adults.^4^, ^5^ Birth represents the main starting point for microbiota establishment at various body sites, including the respiratory tract.^6^, ^7^ The postnatal environment and host properties drive the trajectory of the early‐life respiratory microbiota establishment and maturation.^7^, ^8^, ^9^ Next to bacterial microbiota, the human airways host fungi and viruses that are less abundant compared to their bacterial counterparts and far less studied to date.^10^, ^11^ This review exclusively focuses on the bacterial respiratory microbiome. The constitution of the respiratory microbial community is shaped by the balance of three ecological processes: microbial immigration into and elimination from the community and the relative reproduction rates of community members (Figure 1).^12^ The latter are influenced by the local growth conditions. Additionally, various factors have been shown to influence the developing respiratory microbiome (Figure 1). The establishment of the microbial community is a dynamic process, especially in the first years of life, and the outcome depends on biological phenomena such as immune functions, exposure, mucus transport, and between‐species competition and facilitation. In the following, we will first explore the dynamics of early childhood establishment. Second, we will discuss factors that are thought to influence the microbiome indirectly by influencing biological phenomena, such as immune function and exposure, or directly by elimination (Figure 1). These include endogenous, that is, host‐related factors and environmental factors resulting from external processes and influences. Perturbations of the respiratory microbiota establishment after birth have been associated with increased risk and frequency of respiratory infections and disease severity in early life.^10^, ^13^, ^14^ The neonatal respiratory microbiome not only persistently effects immune maturation,^15^, ^16^ it is also thought to enhance epithelial integrity,^17^ and offers resistance to colonization,^18^ thereby preventing the overgrowth and invasion of potential pathogens.^19^ From a clinical perspective, there is an urgent need to further explore the role of the respiratory microbiome in the neonatal development and identify microbiome‐based strategies for protecting and enhancing human health since early life. This review provides an overview of current knowledge on neonatal respiratory microbiome seeding and succession, key factors driving airway colonization, and implications for infant health. In addition, it highlights the importance of the interaction between the microbiota across different body sites, in particular, the gut and the respiratory tract, for early life development.

## 2 RESPIRATORY MICROBIOME—DIVERSITY AND DISTRIBUTION IN THE RESPIRATORY TRACT

**FIGURE 2:**
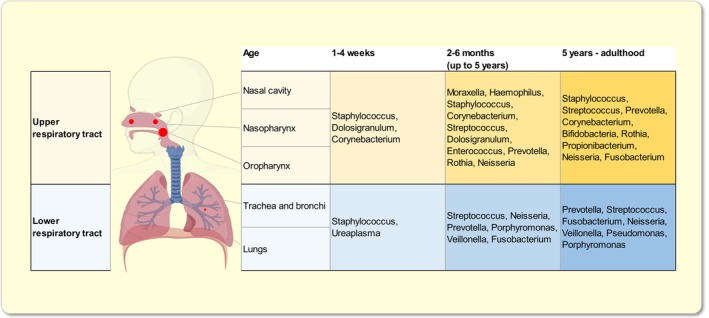
Microbial distribution in the respiratory tract in health at different ages. The size of the red dots visualizes the density of microbiota in the different parts of the respiratory tract (Created in https://BioRender.com).

The respiratory tract consists of a network of organs and tissues whose primary function is the exchange of oxygen and carbon dioxide.^19^ This compartmentalized system divides into the upper respiratory tract, consisting of the nasal cavity, nasopharynx, and oropharynx, and the lower respiratory tract, comprised of larynx, trachea, bronchi, and lungs. Each of these anatomical regions is characterized by specific physicochemical conditions, including pH, relative humidity, temperature, and partial pressure of oxygen and carbon dioxide, which together with anatomical factors including epithelial characteristics and mucosal immune functions shape the microbiota across the respiratory tract.^19^ Although, the density of microbes in the respiratory tract differs by 100‐ to 1000‐fold between the upper and lower respiratory tract,^20^, ^21^ neighboring niches overlap in certain microbial communities (Figure 2).^22^ Bacteria are the major fraction of the complex airway microbial community, represented by the phyla Bacillota, Actinomycetota, Bacteroidota, Pseudomonadota, Fusobacteriota, and Mycoplasmatota, previously referred to as Firmicutes, Actinobacteria, Bacteroidetes, Proteobacteria, Fusobacteria, and Tenericutes.^23^, ^24^, ^25^ The relative abundance of these phyla and their representatives on lower taxonomic levels varies substantially between the different parts of the human airways. The Gram‐positive Bacillota, a major colonizer of the human airways in the upper respiratory tract is represented by aerobic species such as *Staphylococcus*, *Streptococcus*, and *Dolosigranulum*.^5^, ^22^, ^23^ Changes in the oxygen tension from the upper toward the lower airways promotes the colonization of facultative anaerobes such as *Veillonella* and *Lactobacillus* in the lungs.^5^, ^26^, ^27^ Similarly, while Actinomycetota is the second most abundant phyla in the upper respiratory tract, represented by Gram‐positive aerobes such as *Corynebacterium* and *Rothia*, the decrease in oxygen pressure in the lower airways reduces the relative abundance of Actinomycetota species.^5^, ^19^, ^23^, ^25^ Pseudomonadota is the third most abundant phylum, represented by Gram‐negative aerobes such as *Moraxella*, *Haemophilus*, and *Neisseria*, the last primarily found in the lungs.^5^, ^22^, ^23^, ^25^ Fusobacteriota as *Leptotrichia* and *Fusobacterium* are mainly found in the oropharynx.^28^, ^29^ Whereas, the Gram‐negative Bacteroidota is a minor fraction in the upper respiratory microbiome, Bacteroidota species are key members of the lung microbiome comprising anaerobic bacteria such as *Prevotella* and *Porphyromonas*.^5^, ^23^, ^25^ Mycoplasmatota, represented by the single Gram‐negative aerobe *Ureaplasma* is a distinct signature microbiota of the lower respiratory tract in the first weeks of life.^5^, ^30^

## 3 ESTABLISHMENT OF THE RESPIRATORY MICROBIOME

### 3.1 The upper respiratory tract

The colonization of the respiratory tract begins at birth when members of the mother's vaginal, oral, or skin microbiome colonize the neonate upper respiratory tract.^6^, ^31^ During the first weeks of life the nasopharyngeal microbiota develops most rapidly. Initially, it closely resembles the composition of the skin microbiota, likely due to transfer from the mother's skin during breastfeeding, and gradually shifts toward a respiratory microbiota by around 3 months of age.^32^, ^33^, ^34^ *Staphylococcus aureus* emerges as the first core microbe of the upper airways, rapidly followed by colonization and proliferation of the Gram‐positive commensals *Corynebacterium* and *Dolosigranulum*. Species of the genera *Staphylococcus* and *Corynebacterium* are common parts of the human skin microbiome and show a similar temporal pattern of colonization in the nasopharynx with initially high rates that decrease with age.^31^, ^35^, ^36^, ^37^, ^38^ From 6 weeks of age, *Moraxella* spp. rapidly begin to colonize the upper airways and by the age of 3 months dominate the community.^32^, ^35^, ^39^ Species such as *Moraxella*, *Haemophilus*, *Staphylococcus*, *Corynebacterium*, *Streptococcus*, and *Dolosigranulum*, represent 6 relatively simple clusters in the nasopharyngeal microbiome.^35^ After an initial colonization with skin‐dwelling bacteria, a stable colonization with *Moraxella* or *Dolosigranulum* is established and punctuated by transient expansion of *Streptococcus, Moraxella*, or *Haemophilus*, more frequently found during acute respiratory infections.^40^ Accordingly, several longitudinal birth cohort studies located in Europe and America have found that during the first few months of life, the nasopharyngeal microbiome of healthy infants is characterized by a high relative abundance of *Staphylococcaceae*, *Moraxellaceae*, and *Corynebacteriaceae*. Over time, these microbes are gradually replaced by the dominance of *Streptococcaceae* or either *Moraxellaceae* or *Corynebacteriaceae*, along with other minor bacterial families.^7^, ^31^, ^39^, ^41^, ^42^ These species dominate the nasopharyngeal microbiome of infants up to the age of about 5 years. Thereafter, the microbiome gradually resembles that of an adult,^40^ characterized by a higher alpha diversity, a decline in *Moraxella* and *Corynebacterium*, and a reduction in microbial biomass.^43^

### 3.2 The lower respiratory tract

The lower respiratory tract was long considered to be devoid of microbes. However, thanks to advances in high‐throughput molecular sequencing technologies, we now know that the human lung is regularly exposed to microorganisms and their by‐products.^44^, ^45^ Due to the limited accessibility of the lower respiratory tract requiring invasive sampling procedures to ensure controlled sample collection and the low microbial density, especially in healthy infants, our understanding of the neonate lower respiratory microbiome is quite limited.^46^, ^47^ In general, the mature lower respiratory microbiome comprises little biomass and a similar yet distinct microbiota composition compared to the upper respiratory microbiota.^20^, ^21^, ^48^, ^49^ Most studies have used endotracheal aspirates of pre‐term and term‐born infants, who underwent elective surgery or endotracheal intubation for respiratory support, in order to investigate the development of the lower airway microbiome.^30^, ^46^, ^48^, ^50^, ^51^, ^52^, ^53^, ^54^ The overlaps in the composition of the airway microbiota reported between the lower and upper respiratory tract^21^, ^48^, ^49^ support the idea of direct down‐stream mucosal dispersion and micro‐aspiration of content from the upper respiratory tract or even the oral cavity.^21^ In the first weeks of life, bacterial load in the lower respiratory tract is very low with diversity gradually increasing and reaching stability and distinct bacterial community patterns within the first 2 months after birth.^30^, ^55^ In pre‐term infants the early lower airway microbiome is dominated mostly by *Staphylococcus* or *Ureaplasma* spp.^30^, ^51^, ^54^, ^55^ and this pattern remains static over the first month of life.^55^ In contrast, term neonates acquire a mixed microbiota profile faster with a balanced composition including the genera *Streptococcus*, *Neisseria*, *Prevotella*, *Porphyromonas*, *Veillonella*, and *Fusobacterium* ^30^ that resembles the healthy adult lung microbiota.^21^, ^56^ This colonization timing with respect to the switch to a mixed microbiota profile after birth aligns with findings in the upper respiratory tract^31^ and other body sites.^7^

## 4 FACTORS POTENTIALLY SHAPING THE DEVELOPING RESPIRATORY MICROBIOME

The maturation of the respiratory microbiota in the first year of life is driven by the postnatal environment and is associated with colonization events accompanied by appearance and disappearance of certain members of the respiratory microbiota inherited since birth.^32^ Table 1 provides an overview of major factors influencing the developing respiratory microbiome and their impact on certain bacteria at the genus level with the corresponding literature. In this context, we define factors as variables that influence the processes determining the homeostasis of the respiratory microbiome (Figure 1). Moreover, reports such as those on the dependency of respiratory microbiota states on the gestational age suggest that also endogenous, maturity‐related host factors are important determinants of respiratory microbiota development. All these factors might favor the development of a potentially protective or harmful respiratory microbiota. Improving our understanding of the biological relevance of such factors in these complex relationships is therefore mandatory. This task is compounded by the fact that disentangling the effects of different factors on the respiratory microbiome is often difficult as many of them are linked. For example, women who delivered by cesarean section are significantly more likely to discontinue breastfeeding early.^57^ Furthermore, gestational age at birth impacts on breast‐feeding frequency and duration, especially if pre‐term birth is accompanied by a Neonatal Intensive Care Unit (NICU) admission.^58^, ^59^ In addition, according to current knowledge, respiratory tract infections play a dual role in the development of the respiratory microbiome, being both an influencing factor and an associated clinical outcome. This reflects the difficulty of determining the independent contribution of an influencing factor to microbiome composition and function, which would require high‐resolution kinetic studies with simultaneous acquisition of all potentially influencing factors to distinguish confounding factors from true influences. To our knowledge, none of the existing studies has yet fulfilled this task entirely.

**TABLE 1:** Core respiratory microbiota trajectories, influencing factors and diseases associated with aberrant compositional respiratory microbiota states.

Age	Core bacterial colonizers (genera)	Major factors influencing respiratory microbiota formation					Disease associations			References
		Gestational age (preterm vs term)	Mode of delivery (CS vs VD)	Diet (formula vs breast milk)	Antibiotics	Preceding respiratory infections	Respiratory tract infections	Recurrent wheezing and asthma	Broncho‐pulmonary dysplasia	
*Upper respiratory tract*										
1–4 weeks	*Staphylococcus*		↓	↑			↓	↑		[40, 60, 61]
	*Dolosigranulum*		↓	↓	↓		↓	↓		[31, 32, 35, 39, 62]
	*Corynebacterium*		↓	↓	↓		↓			[31, 32, 39, 40, 60]
2–6 months (up to 5 years)	*Moraxella*	↑	↑	↑	↑	↑	↑ (↓)	↑ / ↓		[28, 31, 32, 35, 39, 40, 63, 64, 65, 66, 67, 68, 69, 70]
	*Haemophilus*	↑			↑	↑ (↓)	↑	↑		[28, 35, 39, 40, 62, 63, 65, 66, 67, 69]
	*Staphylococcus*		↓					↑		[61]
	*Corynebacterium*		↓	↓	↓	↓	↓	↓		[28, 31, 32, 39, 40, 60]
	*Streptococcus*	↓		↓ (↑)	↑		↑	↑		[30, 32, 35, 39, 40, 66, 67, 68, 70, 71]
	*Dolosigranulum*		↓	↓	↓	↓	↓	↓		[28, 31, 32, 35, 39, 40, 62]
	*Enterococcus*									
	*Prevotella*			↑				(↑)		[32, 72]
	*Rothia*	↓								[71]
	*Neisseria*	↓		↑		↑				[28, 32, 71]
*Lower respiratory tract*										
1–4 weeks	*Staphylococcus*	↑	↑						↓/↑	[53, 54, 55, 73]
	*Ureaplasma*	↑			↑				↑	[54, 55, 73]
2–6 months (up to 5 years)	*Streptococcus*									
	*Neisseria*									
	*Prevotella*							↑		[72]
	*Porphyromonas*									
	*Veillonella*							↑		[72]
	*Fusobacterium*									

*Note*: Arrows represent reported associations between influencing factors or specific diseases and the relative abundance (↑increased relative abundance, ↓decreased relative abundance) of specific bacterial colonizers; arrows in brackets reflect minor evidence reported by single publications.

Abbreviations: CS, cesarean section; VD, vaginal delivery; vs, versus (compared to).

### 4.1 Gestational age

Pre‐term infants are more likely to fail at developing a healthy microbiome than term‐born neonates. Factors that contribute to this are not only limited to impaired immune regulation and gut immaturity but also include extrinsic prenatal and postnatal factors that disrupt the development of normal flora as maternal infections and premature rupture of membranes, higher likeliness of cesarean delivery, antibiotic exposure, NICU admission, and prolonged hospital stays, increased exposure to respiratory interventions and reduced rates of human milk feeding.^6^, ^9^, ^58^, ^59^, ^71^ The upper airway microbiome of pre‐term infants of <32 weeks of gestation at birth differs from that of term‐born neonates compared at distinct postnatal ages. The nasopharyngeal microbiome of the pre‐term infant at 6 months to ≤2 years is characterized by higher within‐group heterogeneity and an increased relative abundance of Proteobacteria such as *Moraxella* and *Haemophilus* and decreased proportion of Firmicutes compared to full‐term neonates.^63^ Longitudinal metagenomics studies of oropharyngeal swabs in pre‐term compared to full‐term infants indicate that the upper respiratory tract microbiome of pre‐term infants is initially significantly influenced by the hospital environment and therapeutic regimens as mechanical ventilation. After discharge, pre‐term infants lose their hospital‐acquired individual metagenome signatures and develop a common overall taxonomic structure.^72^ However, at least in the oropharynx, the bacterial community structures in pre‐term infants remain different at the age of 15 months compared to healthy full‐term infants that are mainly driven by the number of microbial species (especially the decreased relative abundance of *Streptococcus*, *Rothia*, and *Neisseria*).^72^ It remains to be demonstrated and well controlled for a myriad of other factors that there is a true link between gestational maturity and upper respiratory microbiota development. For instance, the effect sizes of the altered gut microbiota states of pre‐term infants (impacting via the gut–lung axis on the respiratory tract) or the duration of hospitalization might be significantly higher in this respect.

There are only few data on how gestational age may affect the composition of the lower respiratory tract microbiome. Pattaroni et al. found that tracheal aspirates of healthy pre‐term infants are characterized by a prolonged persistence of a diversity‐skewed microbiota after birth dominated by either *Staphylococcus* or *Ureaplasma*, whereas in term babies a mixed, adult‐like anaerobic profile assembled within 7 weeks. Interestingly, the effect of gestational age was stronger than that of other extrinsic factors such as mode of delivery or antibiotic treatment.^73^ The authors concluded that gestational age is the primary driver of variation in microbiota composition during the first 2 months of life. However, the study could not clarify whether the respiratory microbiota differences can indeed be ascribed to gestational maturity deficits or rather link to the hospitalization and restricted environmental exposure of pre‐term infants after birth.

### 4.2 Mode of delivery

Infants born via cesarean section have previously been reported to show alterations in the acquisition and structure of the initial intestinal microbiota^6^ either directly by interrupting the normal transmission route of maternal symbiotic bacteria to infants or indirectly by preventing labor‐associated immune priming of the infant for the postnatal immune adaptation and bacterial colonization.^9^, ^74^ The studies of Bosch et al. suggested that irrespective of the delivery mode *Staphylococcus aureus* is a key member of the healthy early life upper airway microbiome, followed by differentiation toward *Dolosigranulum‐*, *Corynebacterium‐*, *Moraxella‐*, *Streptococcus‐*, and/or *Haemophilus*‐dominated communities.^31^ However, the switch to potentially beneficial nasopharyngeal microbes with regard to respiratory health such as *Corynebacterium*‐ and *Dolosigranulum*‐dominated profiles occurred significantly earlier and prolonged, whereas *Moraxella* spp. enriched later in children delivered vaginally compared to infants born by cesarean section but was also driven by breastfeeding.^31^, ^32^ Ta et al. profiled bacterial nasopharyngeal swabs longitudinally in a birth cohort of primarily full‐term infants and found that cesarean delivery and male sex were associated with a higher relative abundance of *Aerococcaceae* and lower relative abundance of *Staphylococcaceae* in the first months of life.^60^ Another study in healthy term neonates included metagenomics studies next to 16S rRNA profiling of samples from different body sites and found only minor and transient variations of nasopharyngeal microbiota compositions associated with cesarean section while by the age of 6 weeks, the respiratory microbiota composition and function had stabilized with no detectable differences in relation to the mode of birth.^7^ In pre‐term neonates born by elective cesarean section, the oropharynx only initially (first week of life) harbored microbial communities of lower diversity lacking Enterococcus and Gammaproteobacteria compared to infants born by nonelective cesarean section and vaginal delivery. Thereafter, such association could not be detected anymore.^72^

The work of Pattaroni et al. on the formation of lower respiratory tract microbiota revealed that the delivery mode explains a large proportion of microbiota variation in pre‐term, but not term neonates. However, clear species‐delivery mode associations were not apparent.^73^ In ventilated pre‐term infants delivered vaginally, an increase of *Acinetobacter* and *Mycoplasma* in tracheal aspirates was reported, whereas *Staphylococcus* were increased after cesarean section.^55^

### 4.3 Diet

Numerous studies have shown that breast milk feeding modulates intestinal microbiota development either directly through antimicrobial or prebiotic activities or indirectly by influencing the host's immune functions contributing to a protective setting against infections, inflammatory disorders, and allergies.^75^, ^76^ Regarding its influence on the upper respiratory tract microbiota development in healthy infants, a recent study showed that breastfeeding impacts on the microbiota composition in the oropharynx but not nasal cavity, particularly, the relative abundance of *Streptococcus* and *Candida*.^30^ Another study showed that the overall bacterial community composition of the nasopharynx of exclusively breastfed healthy term infants differs significantly at the age of 6 weeks compared to formula‐fed infants.^77^ Breastfed children showed increased presence and relative abundance of the lactic acid bacterium *Dolosigranulum* and *Corynebacterium* and decreased relative abundance of *Staphylococcus* and anaerobic bacteria, such as *Prevotella* and *Veillonella*, compared to formula‐fed infants. *Dolosigranulum* abundance was inversely associated with symptoms of wheezing and mild respiratory tract infections. At 6 months of age, that is, at the time of solid food introduction, associations between breastfeeding/formula‐feeding and nasopharyngeal microbiota composition had disappeared.^39^, ^77^ Additionally, breastfeeding but also vaginal delivery were associated with a late enrichment with *Moraxella* spp.^32^ Contrary, formula‐fed and/or cesarean‐born children had high relative abundances of *Gemella* and *Streptococcus* species from birth, and after the first month of life prolonged predominance of *Neisseria* spp. and (facultative) anaerobes, including *(Allo)Prevotella*, *Ganulicatella*, and *Actinomyces* spp.^32^

In addition, the mode of feeding (oral vs. tube feeding) may have an influence on the respiratory microbiome. Enteral feeding tubes, which are commonly used in the NICU, rapidly develop microbial biofilms that often harbor nosocomial pathogens and increase the risk of intestinal dysbiosis and infection.^78^ The composition of bacterial colonization differs depending on the insertion route, nasogastric vs. orogastric.^79^ However, a small randomized controlled trial of oropharyngeal administration of own mother's milk versus placebo in very low birth weight infants fed by gastric tube showed no effect of the intervention on the oral microbial colonization.^80^ Thought, the significance of this study was limited by the very short follow‐up period of only 21 days. Further studies are required to elucidate any potential effect of the mode of feeding on the respiratory microbiome. Up to date, no studies have addressed the impact of breastfeeding on the lower respiratory tract microbiome. However, maternal diet during pregnancy has been shown to affect the infants’ lower airway microbiota (hypopharynx). A dietary intervention of n‐3 long‐chain fatty acids and vitamin D supplementation to the mother during pregnancy led to changes in the microbiota composition in hypopharyngeal aspirates at the age of 1 month in infants, which in turn was associated with a change in the immune mediator profile. In addition, the maturation of the airway microbial community and the relative abundance of specific bacterial genera were altered. Surprisingly, these alterations were probably mainly driven by a decrease in *Streptococcus* and *Staphylococcus* and a corresponding increase in *Moraxella*, though each of these findings had not reached significance at the genus level.^81^ Whether this observation is directly related to the dietary interventions of the mothers or rather an indirect effect related to changes of the skin and breast milk microbiota of the mothers has not been addressed in this study.

### 4.4 Antibiotics

Antibiotic treatment is an important factor known to modify microbiota composition and diversity. In healthy infants, antibiotic usage during the 4 weeks prior to sampling was associated with higher relative abundances of *Haemophilus*, *Streptococcus*, and *Moraxella* and lower relative abundances of *Corynebacterium and Dolosigranulum* in the upper respiratory tract (nasopharynx).^32^, ^35^ Therewith in line, another longitudinal study in children with antibiotic exposure during the age of 0–2 months found *Haemophilus* increasing with age among nasal microbiota in the first 2 years of life, whereas in children without early‐life antibiotic exposure, *Dolosigranulum* increased with age.^62^ In a large Finnish population‐based birth cohort study, repeated antibiotic treatments during the first 11 months of life were associated with a nasal microbial profile with early *Moraxella* sparsity until 13 months of age followed by a rapid rise thereafter.^64^, ^82^ In pre‐term infants, antimicrobial therapy during the postnatal hospital stay led to a significant reduction in the diversity of high and low abundance taxa in the oropharynx by the age of 9 months. But this effect vanished by the age of 15 months.^72^

In the lower respiratory tract of pre‐term infants, antibiotics did not affect alpha diversity in successfully sequenced tracheal aspirate samples collected during the first 28 days of life, suggesting that, rather than suppressing specific species, they were suppressing bacterial growth globally. Only a marginal increase of Mollicutes (*Ureaplasma* and *Mycoplasma*) was detected compared to the no‐antibiotic group.^55^ Also in the study of Pattaroni et al., antibiotic treatment did not significantly affect the composition of the lower airway microbiome in both pre‐term and term neonates.^73^

In addition to antibiotics, other medications such as postnatal steroids potentially influence the taxonomy and longitudinal development of microbiomes after birth. Studies in adult patients and mice have shown that local or systemic corticosteroid use is associated with changes in the airway microbiome.^83^, ^84^, ^85^ Grier et al. showed that steroids used for the prevention or treatment of bronchopulmonary dysplasia significantly altered the intestinal relative abundance of *Bifidobacterium* in a cohort of pre‐term infants.^86^ Therefore, an effect of steroid treatment on the developing respiratory microbiome in neonates is conceivable but, to our knowledge, has not yet been demonstrated.

### 4.5 Early‐life infections

Respiratory infections in early life have been shown to affect the composition of the upper respiratory tract microbiome not only acutely but also its development in the long run. Teo et al. reported a higher relative abundance of *Moraxella* and reduced relative abundance of *Dolosigranulum* or *Corynebacterium* in nasopharyngeal samples of infants and young children following increased numbers or respiratory infections.^35^ However, this study did not clarify whether the microbiome has been altered already before and thus rather predisposed to respiratory infections than being a sequela. De Steenhuijsen Piters et al. recently revealed that early asymptomatic viral infections subsequently led to altered microbiota dynamics in the nasopharynx including early enrichment of *Moraxella* and *Haemophilus* spp., which in turn was related to a higher number of subsequent viral respiratory tract infections. The latter is in so far surprising as asymptomatic viral infections increased the overall tonus of interferon activity in the nasopharynx.^65^ Korten et al. found that only symptomatic but not asymptomatic human rhinovirus infections in healthy full‐term infants during the first year of life were associated with acute changes in the nasal microbiota, primarily characterized by a decrease in microbial diversity. In infants with more frequent symptomatic human rhinovirus infections, the lower bacterial diversity persisted throughout the first year of life.^87^ In a cohort of infants born between 32 and 35 weeks of gestation, respiratory syncytial virus (RSV) infection during infancy was associated with a lower relative abundance of *Haemophilus* spp. at the age of 6 years and a significantly higher relative abundance of *Moraxella* and *Neisseriaceae* than children without RSV infection during infancy.^28^ To our knowledge, there are no data available on the impact of early respiratory tract infections on the developing lower respiratory tract microbiome.

### 4.6 Other factors shaping the respiratory microbiome

Other important determinants of respiratory microbiota composition, in particular in the upper respiratory tract, include co‐habiting with siblings and daycare attendance. Both are known risk factors for respiratory tract infections, which might be explained by a premature enrichment of *Moraxella* spp., higher relative abundance of *Haemophilus* spp. and lower relative abundances of species such as *Corynebacterium* and *Staphylococcus*.^32^, ^35^, ^60^ Furthermore, the country in which the child was born significantly influences the overall nasal microbiome composition^30^ as well as the season of birth. At 1 month of age, summer‐born infants exhibited greater bacterial richness and a higher abundance of specific bacterial profiles representing Gram‐negative alpha‐Proteobacteria and Gram‐positive Bacilli in the nasopharynx.^88^

## 5 ASSOCIATIONS BETWEEN THE NEONATE RESPIRATORY MICROBIOME AND RESPIRATORY DISEASE MANIFESTATIONS

The respiratory microbiota composition in infants and children has been associated with the manifestation of different respiratory diseases whereby most data are based on associations with the nasopharyngeal microbiota. Moreover, there is multiple experimental evidence in mice that interactions between the colonizing respiratory microbiota and the airway mucosa influence the structural development of the respiratory tract and mucosal immune system in the postnatal period and thus shape the infants' susceptibility to respiratory disease.^15^, ^16^, ^89^, ^90^, ^91^, ^92^, ^93^ Collectively, these findings are highly suggestive of a considerable interdependency between respiratory microbial communities and the host's respiratory mucosal immune system that needs to be understood for preventive or therapeutic exploitation. Table 1 provides an overview of differences in the relative abundance of bacteria of the developing respiratory microbiome in the neonatal period and in infancy that are associated with respiratory diseases in infancy and childhood and the corresponding literature.

### 5.1 Respiratory tract infections

One of the first longitudinal studies on upper respiratory microbiota profiles of healthy children during the first 2 years of life observed that a delayed and low relative abundance of *Moraxella, Corynebacterium*, and *Dolosigranulum* and a high relative abundance of *Haemophilus* or *Streptococcus* was positively associated with higher rates of respiratory infections.^39^ A longitudinal study in healthy infants and children showed that infants with a higher predisposition to respiratory tract infections in the first year of life had an aberrant microbial developmental trajectory from the first month of life compared to infants with rare airway infections. Here, the altered nasopharyngeal microbiota was described to lack *Corynebacterium* and *Dolosigranulum* spp. establishment and transit prematurely from *Staphylococcus*‐dominated toward a *Moraxella*‐dominated profile.^32^ Accordingly, Lapidot et al. reported from a longitudinal Zambian mother–infant cohort that infants who developed a lower respiratory tract infection had nasopharyngeal dysbiosis before infection, in most cases as early as the first week of life. Dysbiosis was characterized by the presence of *Novosphingobium*, *Delftia*, high relative abundance of *Anaerobacillus*, *Bacillus*, and low relative abundance of *Dolosigranulum*, compared to the healthy controls. Interestingly, mothers of infants with lower respiratory tract infection also had low relative abundance of *Dolosigranulum* in their nasopharyngeal samples 1 week after birth compared to mothers of infants that did not develop lower respiratory tract infections.^94^ Similarly, in a longitudinal study of the nasal microbiota of infants from 3 weeks to 18 months of age, the microbiota composition differed in infants with rhinitis with concomitant wheeze compared to healthy controls prior to the infection episodes. While microbial diversity increased over the period of 18 months of life in the control infants, the diversity decreased in the infants with rhinitis. An increase in the relative abundance of species belonging to the families *Oxalobacteraceae* and *Aerococcaceae* was associated with rhinitis and concomitant wheeze, while *Corynebacteriaceae* and early colonization with *Staphylococcaceae* during the first 9 months was associated with the controls.^60^

Several studies suggest that the respiratory microbiota influences the development of the host's immune systems, however, the extent of their interrelatedness is incompletely understood. Especially, the chicken‐and‐egg question remains unresolved. In the Copenhagen Prospective Study of Asthma in Childhood (COPSAC) birth cohort, colonization of the hypopharynx of neonates with *Moraxella catarrhalis* and *Haemophilus influenzae* induced a mixed T helper (Th) cell type 1/Th2/Th17 response with high levels of IL‐1β, TNF‐α, and MIP‐1β in the airway mucosal lining fluid, whereas *Staphylococcus aureus* colonization promoted a Th17‐related cytokine.^95^ A longitudinal comparative study of pre‐term and full‐term infants also reported on a tight co‐evolution between T‐cell maturation and respiratory microbiota community structure with early atypical or asynchronous immune and microbiota features in infants predicting persistent respiratory disease.^96^ A cross‐sectional study of colonization and immune system maturation in the lower airways of pre‐term and term‐born infants observed that the IgA1 protease activity as a measure of virulence potential of bacterial communities correlated with a significant enrichment in lung gene expression related to the IgA production pathway, while both correlated with gestational age.^73^ These data suggest an intense cross talk between the airway microbiota and the host immune system. However, it remains unclear whether microbial gene expression drives the immune response or whether the immune response drives the microbiota composition. However, it is probably most similar to an infinite circle of repeating influence rather than a one‐off succession as the chicken‐egg principle would suggest.

An important factor for the interplay between microbiota development and risk for subsequent disease is the timing of colonization succession. Several studies in mice have demonstrated that timely cues from the respiratory microbiota are required for healthy neonatal immune development.^15^, ^16^ In human infants, though almost all children transit to a *Moraxella*‐dominated microbiota by the age of 3–6 months, particularly premature colonization with *Moraxella* spp. and/or *Streptococcus* were shown to induce a mixed inflammatory immune response of the airway mucosa^95^ and associate with a higher risk of earlier‐onset respiratory tract infections and wheezing.^35^, ^40^, ^65^ This phenomenon has previously been described as the neonatal window of opportunity, in which the establishment of microbiota and maturation of the neonate's innate and adaptive immune system takes place and ensures the development of long‐term immune homeostasis.^97^

### 5.2 Recurrent wheezing and asthma

As early as 2007, culture‐based analyses of hypopharyngeal aspirates in a prospective cohort study showed that neonates colonized with *Streptococcus pneumoniae*, *Haemophilus influenzae*, and/or *Moraxella catarrhalis* were at increased risk for recurrent wheeze and asthma early in life.^66^ Since then, the association of distinct colonization patterns of the upper respiratory tract during the first 5 years of life, such as *Moraxella*‐, *Haemophilus*‐, *Streptococcus*‐, and also *Staphylococcus*‐dominant microbiome with increased risk of recurrent wheezing and asthma has been confirmed by culture‐independent techniques in multiple studies.^40^, ^61^, ^67^, ^70^ In a large Finnish population‐based birth cohort study, the investigators had a strong focus on the dynamics of the nasal respiratory microbiota development. Here, an early *Moraxella* sparsity profile (during months 2–13) with subsequent rise as well as persistently high relative *Streptococcus* abundances were associated with a significantly higher risk of asthma at 7 years of age compared with a persistent *Moraxella* dominance profile.^89^ A subanalysis of the MAKI trial found for recurrent wheezing a positive association with relative *Haemophilus* abundance and also negative association with the relative abundance of taxa such as *Moraxella*, *Corynebacterium*, *Dolosigranulum*, and Staphylococcus at 6 years of age.^28^ However, it remains yet elusive whether the risk of chronic wheezing and asthma is rather indirectly associated with the respiratory microbiota structure and primarily determined by the number and severity of lower respiratory tract infections in the first year of life.^40^

In a large Danish population‐based birth cohort, distinct microbiota compositions in hypopharyngeal aspirates of healthy 1‐month‐old infants, specifically the relative abundances of the taxa *Veillonella* and *Prevotella*, were associated with later asthma. This asthma‐associated composition furthermore correlated with lower levels of topical pro‐inflammatory airway immune mediators (IL‐1β and TNF‐α) and higher levels of monocyte and T‐cell recruiting chemoattractants (CCL‐2 and CCL‐17). This could indicate a delayed overall induction of microbiota‐driven immune stimulation, which may predispose to long‐term immune dysregulation, low‐grade inflammation, and immune‐mediated noncommunicable diseases later in life.^98^

One of the rare studies trying to address the chicken‐and‐egg question showed that prenatal maternal low IFN‐γ:IL13 secretion during the third trimester of pregnancy is associated with an impaired responsiveness of the neonate's cord blood mononuclear cells to microbial products. Affected infants exhibited a distinct pattern of upper airway microbiota development characterized by early‐life colonization with *Haemophilus* that later transitioned to a *Moraxella*‐dominated microbiota and developed asthma during childhood.^69^ These data suggest that perturbations of the neonate's immune programming can lead to altered patterns of airway microbiome colonization. However, the mechanisms underlying this observation are only poorly understood.

### 5.3 Bronchopulmonary dysplasia

Several authors have studied the respiratory microbiome and its impact on disease characteristics in preterm infants with bronchopulmonary dysplasia (BPD), the most frequent complication of extreme pre‐term birth. Multiple factors contribute to the development of BPD with pulmonary inflammation being the common constant that leads to a BPD phenotype.^99^ In aspirates of the lower respiratory tract of pre‐term infants, episodes of increases in IL‐6 and IL‐8 corresponded to increased bacterial load and to the presence of predominant operational taxonomic units such as *Acinetobacter*, unclassified *Enterobacteriaceae* and *Mollicutes* including *Mycoplasma* and *Ureaplasma*.^55^ Therefore, it seems plausible to suspect disturbances in the respiratory microbiome composition causing or accompanying the altered postnatal pulmonary immune development. BPD has been associated with a less diverse lower respiratory tract microbiome, presence of *Ureaplasma* spp., and reduced *Lactobacillus* detection, whereas the associations with the relative abundance of *Staphylococcus* are contradictory.^46^, ^53^, ^54^ In a follow‐up Lal et al. reported in a metagenomic and metabolomic follow‐up study that the reduction in *Lactobacillus* and relative increase in *Enterobacteriaceae* was accompanied by an altered airway metabolome characterized by enrichment of metabolites involved in fatty acid activation that might contribute to airway inflammation in BPD‐predisposed infants.^100^ Xu et al. determined airway microbiome and metabolome signatures in parallel in tracheal aspirates of mechanically ventilated pre‐term infants at birth and day 7 of life. They found a decreasing diversity of the airway microbial community over time in infants developing BPD compared to non‐BPD infants. Moreover, they revealed a close relation between the relative abundance of *Stenotrophomonas* and its metabolite sn‐glycerol 3‐phosphoethanolamine and the occurrence and severity of BPD.^101^ In a further study examining the upper airway microbiome of pre‐term infants at the age of 1 and 3 weeks using nasal swabs they found an increased relative abundance of *Prevotella* and a decreased relative abundance of *Caulobacter* in infants that were later diagnosed with BPD.^102^

Overall, the current data on potential respiratory microbiota–BPD associations are still quite leaky and do not allow identifying clear linkages. In particular, it remains unclear whether the aberrant microbiota composition drives the inflammatory immune response or whether an impaired postnatal immune development alters the respiratory microbiome trajectory.

## 6 GUT–LUNG AXIS

Despite the fact that the gut and the respiratory tract are separate organs with different functions, they have the same embryonic origin and accordingly share structural similarities.^103^ This suggests a potential interaction between the two organs in terms of a shared mucosal immune system and is referred to as the gut–lung axis.^104^ Accordingly, the gut–lung axis has been proposed as a potential target to treat lung disease.^105^, ^106^

The postnatal environment shapes the composition and functionality of the gut microbiome and specific changes in gut microbiota induced by delivery mode, feeding, and antibiotic exposure have been clearly linked to the protection against or predisposition toward respiratory disease in childhood.^107^, ^108^, ^109^, ^110^ The early life microbiota of babies delivered by cesarean section is characterized by the prevalence of bacteria from the phyla Proteobacteria such as *Klebsiella* and *Enterococcus* species marked by a high potential of multi‐drug resistance^111^ and associated with a higher incidence of respiratory infectious events later in life.^108^ Furthermore, cesarean section leads to delayed seeding of the neonate gut with species such as *Bifidobacterium* and the absence of *Bacteroides* species.^108^, ^112^ *Bifidobacterium* and *Bacteroides* species are important for the digestion of prebiotic human milk oligosaccharides in the neonatal gut and the production of short‐chain fatty acids (SCFAs) including acetate, propionate, and butyrate.^113^, ^114^ SCFAs circulate through the bloodstream and stimulate immune responses in the lung.^115^, ^116^ In particular, microbially produced acetate has been shown to protect against RSV infections in mice by improving type 1 interferon responses and increasing interferon‐stimulated gene expression in lung epithelial cells through a mechanism involving GRP43 activation.^117^ Similarly, propionate, a key metabolite from the fermentative activity of *Bacteroides* species in the gut, has been shown to play a key role in early life protection against bronchiolitis.^118^ Furthermore, decreased levels of microbial‐produced intestinal SCFAs as pentanoate and hexanoate were reported to lead to an altered pulmonary type‐2 innate lymphoid cell function and an increase in first‐breath‐ and infection‐induced inflammation.^119^ Several studies in mice have reported a protective role of the gut microbiota in bacterial and viral respiratory infections by influencing alveolar macrophages and their responsiveness^120^, ^121^ and by increasing the pulmonary granulocyte‐macrophage colony‐stimulating factor (GM‐CSF) production.^122^ Moreover, commensal‐mediated regulation of IFNβ enhances natural resistance to virus infections as influenza in mice.^123^ In human infants, associations between specific gut microbial profiles and respiratory diseases have been identified. RSV disease severity in infants hospitalized with RSV was associated with differences in alpha and beta diversity of the gut microbiota at the time of hospitalization.^124^ The increased risk of childhood asthma for infants born via cesarean section has been linked to a metabolic profile indicative of perturbed gut microbiota.^125^ Furthermore, a less‐diverse meconium metabolome and reduced gut microbiome maturation during infancy have been associated with the development of allergic sensitization.^126^ These findings demonstrate that particularly the functional state of the gut microbiome influences the respiratory immune system and susceptibility to respiratory tract infections. If the function matters more than the composition of the gut microbial community it is intriguing to speculate that the metabolome might be the superior factor driving the host's immune responses including those in the airways and the lungs. Because the gut microbiome is the human's largest microbial habitat, it might play the superior role in educating and directing pulmonary immune responses and explain why there are still many remaining uncertainties regarding the role of the respiratory microbiome in infectious and noncommunicable respiratory diseases.

### 6.1 Limitations in lung microbiome research

Research into the respiratory microbiome, particularly of the lower respiratory tract, is limited by various factors including anatomical conditions and limited access to samples, especially from healthy subjects. Specimens obtained by bronchoscopy are at risk of contamination during the obligatory passage of the bronchoscope through the upper respiratory tract. Therefore, the procedure should be performed by an experienced bronchoscopist and results should be interpreted with caution.^20^ Furthermore, the low microbial density in the lower respiratory tract requires the use of appropriate molecular techniques and the inclusion of positive and negative controls in test series in order to reduce artifacts and misinterpretation. In addition, the viability of most of the bacteria obtained can be verified using advanced cultivation techniques.^127^

## 7 CONCLUSIONS

Despite increasing knowledge of the respiratory microbiota development in human neonates, our understanding of the cross talk between respiratory microbial communities and the host is still limited leaving many open questions regarding the causative role of the respiratory microbiota in respiratory diseases. Even certain associations such as, for example, *Moraxella* colonization of the upper respiratory tract with recurrent wheezing and asthma is not entirely clarified. However, several studies consistently reported of associations between respiratory illness as recurrent wheezing and upper respiratory tract infections and shifts of microbiota abundances in the upper respiratory tract during the first month of life, specifically a reduction of *Corynebacterium* and *Dolosigranulum* as seen after cesarean section, antibiotic therapy, or at the absence of breastfeeding or an increase of *Haemophilus* and *Streptococcus* as provoked by antibiotic therapy (Table 1). Due to the scarcity of data, the role of the lower respiratory tract microbiota for the development of respiratory health and the influence of potentially impacting endogenous and environmental factors is particularly difficult to evaluate. This becomes especially obvious for potential microbiota associations with BPD in pre‐term infants. Additional research in the neonatal period and methodical optimizations are essential to better understand the early colonization of infants' airways.

With respect to host–microbiota interactions, it also remains entirely unclear whether immunological host conditions precede a specific colonization trajectory or whether the microbiome composition determines the host immune development—the old chicken‐and‐egg‐question. Moreover, even though distinct microbiome compositions and developmental trajectories in the various parts of the respiratory tract have been described, overall, metagenomics data allowing evaluation of the functional respiratory microbiota state are lacking. In addition, very little is known about the absolute abundance of microbiota components, that is, the actual number of cells, in the respiratory tract as most studies examine relative abundances and diversity, limiting our understanding of a quantitative effect.

Various studies have shown that the functional state of the gut microbiota has a major impact on the respiratory immune system and the susceptibility to respiratory tract infections. As the gut microbiome represents the human's largest microbial habitat and given the uncertainties about the role of the respiratory microbiota, it is appealing to speculate that the gut microbiota might have a superior importance in directly or indirectly directing pulmonary immunity than the respiratory microbiome.

Overall, our understanding of the exact mechanisms of respiratory microbiome–host interactions in humans are still fragmentary. Further research is needed to better understand how the respiratory microbiota influences the development of the host's immune systems and vice versa in order to translate our knowledge into microbiome‐based strategies for protecting and enhancing human health since early life.

## AUTHOR CONTRIBUTIONS

**Sabine Pirr:** Investigation; funding acquisition; writing – original draft; visualization; writing – review and editing. **Maike Willers:** Investigation; writing – original draft; writing – review and editing. **Dorothee Viemann:** Investigation; writing – review and editing; visualization; validation; supervision; funding acquisition; writing – original draft.

## FUNDING INFORMATION

This work was supported by grants from the Federal Ministry of Education and Research (BMBF) to SP and DV (PROSPER; 01EK2103B and 01EK2103A, respectively) and the Deutsche Forschungsgemeinschaft (DFG, German Research Foundation) to SP (PI 1512/1‐3) and DV (VI 538/6‐3 and VI 538‐9‐1). Further support was provided by the DFG SFB 1583/1 (“DECIDE”) project number 492620490 to and the DFG TRR 359 (“PILOT”) project number 491676693 both to DV, and the DFG Germany's Excellence Strategy—EXC 2155 “RESIST”—Project ID 390874280 to DV and SP. MW was supported by the Hannover Biomedical Research School (HBRS) and the Center for Infection Biology (ZIB). The funding sources had no impact on the writing of this manuscript.

## CONFLICT OF INTEREST STATEMENT

Nothing to disclose.

## Data Availability

Data sharing is not applicable to this article as no new data were created or analyzed in this study.
